# Collectanea

**Published:** 1826-10

**Authors:** 


### COLLECTANEA.

Floriferus, ut apes, in saltibus omnia libant,

Omnia nos, itidem, depascimur aurea dicta.

### PATHOLOGY.

*Communication of Hydrophobia.*—Numerous experiments made by the Professor of Surgery of the great hospital of Florence, (M. Betty), and which are published in the Memoirs of the Imperial and Royal Academy, establish the result that sheep, and other animals of the same species, cannot transmit the hydrophobia which has been communicated to them by a mad dog, even if they should themselves die of the disease; that the virus, which kills them, loses its contagious qualities in passing into these animals; that their saliva, or any other liquid or solid inoculated from them into others, produces no evident effect; and that it is the same if the flesh of those animals, who may have died rabid, is eaten by man,—it is quite harmless. These facts are stated to tranquillise the minds of those who, without being aware of it, may have touched any part of a rabid animal, or may have eaten of its flesh. (*Annali Universali.*)

*Phlebitis.*—A communication by M. Gendrin, in a recent Number of the Revue Medicale, contains some observations on this subject.

Inflammation of veins is sometimes produced by the operation of phlebotomy, made with an instrument that is dirty, rusted, or blunt. This inflammation always has its seat in the part of the vessel above the incision: it ascends often in a progressive manner, following the direction of the course of the blood, even in the larger trunks. Intense phlebitis has been produced by bleeding with a lancet impregnated with the vaccine virus. We have seen an inflammation of the cephalic vein, through its whole extent, come on under the following circumstances: The patient was very fat, the operator inexperienced; the opening of the vein was small, and the escape of the blood impeded by the subcutaneous fat. The operator rubbed the arm to assist the exit of the blood, and in doing so repeatedly ruffled the lips of the wound: the consequence was the formation of a small abscess at the part punctured, and, after a time, inflammation of the vein showed itself, and progressively extended to the trunk of the axillary. Serious symptoms came on, and there formed an abscess of three or four inches in extent in the course of the vessel, whose inflamed and dilated coats contained the product of the suppuration; but, what is remarkable, the phlebitis did not commence till after the subcutaneous abscess was formed at the bend of the arm, where the puncture was made.

If veins can thus inflame after the simple incision of phlebotomy, it is more likely to happen in more complicated and extensive operations on them, and it has been remarked after ligatures or sections of the venous trunks, for the cure of varices. Travers cites several such examples; Osiander and Meckel have seen inflammation of the umbilical veins, and, in consequence, of the vena portæ, caused by ligature on the umbilical cord.

We have dissected with care three subjects who died from the consequences of lithotomy: one had been cut by the high operation, the other two by the lateral. We found inflammation in all the abdominal veins, even up to the cava: the veins of the bladder and the hypogastric veins were inflamed, in two of the subjects on whom the lateral operation had been performed; in the third, the left hypogastric vein and the vena cava ascendens were inflamed; there existed also intense peritonitis; the two others offered traces of violent cystitis and enteritis, with slight pneumonia of the left side. We are convinced that persons operated on for the stone often sink from the spreading of inflammation to the veins; and, perhaps, also almost all who undergo severe operations, particularly amputations.*

It is interesting to remark, that the phlebitis following wounds seldom shows itself till suppuration is established in the wound, as occurred in the case of the woman mentioned above, where the cephalic vein inflamed after an abscess had formed at the bend of the arm. This circumstance is still more curious as regards the etiology of inflammations; for it cannot be doubted, after the facts

* See observations on a case of Inflammation of the Veins after Amputation, by Mr. Guthrie, in the Number of this Journal for July.

related by M. Ribes, in the third volume of the Mémoires de la Societé d'Emulation, and in the eighth volume of the Revue Medicale, 1825, and after several other similar facts collected by different observers, that there takes place, by the small branches of the veins, an absorption of pus with which the edges of wounds become infiltrated, or which is deposited in abscesses. In the body of a man, forty-nine years old, who died from phlegmonous erysipelas, occupying all the fore part of the thigh and a part of the integuments of the abdomen, besides abscesses, containing pus and purulent matter, in the cellular tissue, we found pus, mixed with a reddish serosity, in the trunk of the internal saphena vein. It is remarked also, in persons dying of small-pox, that the venous branches coming from parts of the skin covered with confluent pustules, are generally filled with that fluid, and often inflamed. The inflammation of veins originating in diseased tissues, already remarked by Mr. Travers, is now an attested fact: it does not constantly occur, but very frequently. Can the propagation of inflammation to the veins be attributed to the absorption of pus, by the minute branches of veins in the diseased tissues, as it is formed in the lips of the wound?

Inflammation of veins shows itself sometimes without any such causes as those now detailed; as in the abdominal, and particularly the hypogastric veins, in parturient women. It is known that some modern physicians have pretended to explain all puerperal œdemas by this cause. We can only attribute these examples of phlebitis to the singular disposition to inflame that gestation and childbirth leave in all the abdominal viscera; a disposition by which the frequency of peritonitis and enteritis in parturient women, has been sought to be explained. The inflammation of veins is in this case more readily imagined than that of the other abdominal organs, on account of the great change that takes place during gestation, and also during the delivery, in the circulation of blood in the veins of the uterus, fallopian tubes, and ovaries.

The veins which inflame most frequently, *cæteris paribus*, are those of the limbs, and particularly of the lower limbs; those of the pelvis may be reckoned next. There are few examples of phlebitis in the thorax, and still fewer in the brain: perhaps this may arise from the little attention generally given, in opening bodies, to the examination of the vessels. In calling attention to the small number of cases of cerebral phrenitis known, perhaps we may induce practitioners to examine the vessels, in opening the bodies of individuals who have died of diseases of the brain, and verify the inductions that the few facts known allow us to draw.

M. Ribes has related, in the Revue Medicale, July 1825, a case of inflammation of the superior longitudinal sinus, in which he discovered, on opening the body, a considerable thickening of the coats of the venous canal, where the inner surface was lined with a false membrane. The superior cerebral veins, which opened into this sinus, were varicose; a part of this vessel, as well as the left lateral sinus, was obliterated by a fibrous matter, very firm, and evidently organised; a cancerous tubercle existed in the centre of the right hemisphere. This phlebitis was evidently chronic, and did not appear to us to have any connexion with the cancerous disease of the right hemisphere. The interruption of the circulation in the sinus and veins which terminate in it, was not in this case followed by hemorrhagy; but we must remark, that this obstruction of the vessels was here evidently chronic, and it well demonstrated that disorders, which are not quickly formed, are not followed by the same effects as those where the progress is rapid. We must consider as the result of that obstruction the considerable dilatation of the longitudinal sinus, as well as the varicose dilatation of one of the veins which opened into the sinus. This dilatation was so little recent, that M. Ribes remarked on the left parietal bone a deep groove which received this vein, and a remarkable augmentation of the depth and breadth of the longitudinal groove in the back part of the cranium.

---

### PRACTICAL MEDICINE.

*On the Employment of Camphor in Acute and Chronic Rheumatism.*—It must be interesting (says M. Dupasquier,) to learn that there is a method which is almost sure to stop the progress of acute rheumatism, a disease which is generally much prolonged, in spite of the employment of the most rational means. This becomes still more interesting at present, when the "medicine expectante" in diseases, the nature of which is well known, has fallen into just discredit, and when it is acknowledged how dangerous it is, in such a case, to wait for the efforts of an imaginary "vis medicatrix." This method, which consists in applying camphor chiefly under the form of vapour, although little known, is not new: Dr. Amable Cheze, at Chalons sur Saone, appears to have been the inventor of it. This physician had already employed camphor with success in tetanus, and, thinking he saw an analogy between it and rheumatism, was induced to try it in the latter. In his Thesis (1808), "Propositions sur le Rheumatism aïgu et chronique, &c.," Dr. Cheze sets forth his ideas on the employment of camphor in rheumatism; since then, however, it has scarcely been noticed. M. Dupasquier has tried it in several cases, and constantly with success after the first or second fumigation. He cites five cases where purgatives were decidedly useful: the second of them is interesting, not only from the good effects of the remedy, but by affording a contrast to the ordinary mode of treatment.

M. G—, aged forty-four, of sanguineo-nervous temperament, subject to long and violent attacks of acute rheumatism, has felt, since the beginning of June 1825, some stiffness in the joints of the limbs, and in the muscles of the back part of the neck. On the 5th of the same month he went into the country; while the weather was cold and rainy, he sat down on a wet stone, and walked on the moist grass. In the evening he drank, on going to bed, a slightly diaphoretic infusion; but, in spite of this precaution, the next day (June 6th) he was seized with violent pains in the hip-joint and knee of the right side. To these pains were added the following symptoms:—frequent pulse, but neither strong nor full; tongue loaded; severe headache; constant diaphoresis. A camphor fumigation was ordered, during which the perspiration was very slight. Low diet, and infusion of balm for drink. He passed a bad night.

7th.—Much fever: the pains are very acute, and in the same joints; the least movement makes the patient cry out.—Another fumigation: this was stronger of camphor than the last, and produced an abundant flow of perspiration. The pains ceased instantly. The patient, covered with a blanket, was put to bed, and continued to perspire copiously for half an hour: he was quiet for some hours, and slept a little. The pains returned in the afternoon, but were trifling: another fumigation dispelled them. In the night they suddenly returned, and fixed themselves in all the joints of the left leg.

8th.—The patient has suffered much. Fumigated: was much relieved, and could even walk to his bed. He slept some hours, and again used the camphor vapour in the afternoon, and passed an excellent night.

9th.—All febrile symptoms diminished; pains almost gone.—Let him fumigate twice; low diet and barley-water. Passed a good night.

10th.—More fever and more pain; bowels open. Two fumigations, and diet, &c. as yesterday.

11th.—Quite free from pain and fever. Two fumigations.

12th.—Convalescent. He went on daily improving till quite well; but continued the fumigations as a precautionary measure.

This gentleman, the subject of the foregoing case, had been treated the year before, for a similar attack of rheumatism, on the antiphlogistic plan. Repeated applications of leeches on all the diseased joints only temporarily relieved the pain, without removing the disease, which, in spite of every means, lasted more than two months, and was only cured, during the third month, by employing aromatic baths, blisters, purgatives, and afterwards the mineral waters of Aix.

How does camphor act in thus stopping the progress of rheumatism? Is it from its diaphoretic power only? or is it by a particular sedative action that it instantly calms the pains? or, rather, may it not be by an union of these two effects that its remarkable energy is to be explained? M. Dupasquier thinks that this medicine destroys the inflammatory state by a powerful revulsion,—that is, by producing perspiration; and afterwards by its absorption through the skin and pulmonary organs, by acting on the general and first cause of the disease as a sedative.

To exemplify the power of camphor as a sedative, M. D., in a note, states that a patient who had employed with success camphor fumigations for chronic rheumatism, had shortly after severe pains in the left shoulder-joint. As he had great repugnance to renew the fumigations, a little bag of camphor was put under the armpit of the side affected: the absorption was rapid; in half an hour he felt a kind of numbness in the joint, and the pain soon left it. He frequently renewed this experiment, with the same success. With regard to the manner of administering the camphor in rheumatism, it may be given internally or in frictions, or by putting it immediately in contact with the skin, either in powder or vapour; and this last method, uniting the two modes of action of the medicine, is greatly to be preferred. M. Dupasquier has always used it in vapour only, although he thinks there may be cases where it might be advantageously used internally at the same time. There are circumstances, too, that would indicate general bleeding before using the fumigations. The best mode of applying the fumigations, is by an apparatus invented by M. Rapau, consisting of a particular kind of box. Where patients cannot afford this, however, they may be placed on a chair, over a small furnace, with a large blanket thrown round them, reaching to the ground, and drawn close round the neck: a tea-spoonful of powdered camphor is thrown every five minutes on a metal plate covering the furnace. The medicine rapidly volatilises, and the parts of the body with which it comes in contact are speedily covered with sweat. The fumigation may be continued three-quarters of an hour, or an hour. When the operation is finished, the patient is wrapped up in the blanket, and put to bed, where he will continue to perspire for some hours. Half an ounce of camphor is usually enough for a fumigation, but it may be carried to a much greater extent without inconvenience: one patient used four ounces at once without any unpleasant accident. When patients cannot be moved out of bed, the bed-clothes may be raised around them, and the camphor volatilised by means of a warming-pan moved up and down in the bed. While undergoing fumigation, the patient should drink some mildly diaphoretic drink. The number of fumigations in the day must depend on the violence of the pains, and strength of the patient: when he is strong, and suffering much, they may be used three and four times daily. It is always necessary to continue their use for at least a week after all pain has disappeared. (*Revue Medicale.*)

*Case of Rheumatism of the Heart, treated by Acupuncture.* By M. Peyron.—A woman, eighteen years of age, of good constitution, and of nervous temperament, having lived for some years in a very damp house, began to feel pains in her arms, and afterwards in her legs: they were not fixed, and were at first considered as *growing* pains, but they soon shewed the true nature of the disease. Various means were unsuccessfully employed while she remained in the same house, but, as soon as she had quitted it, the pains diminished in intensity, and then disappeared. She was next attacked with severe pain in the region of the heart. This pain was not continued, but frequently recurred, not only from changes of weather, but also from any lively emotion, and sometimes remained for days: it was accompanied with palpitations, which daily became stronger, and at times was accompanied by contraction of all the muscles, so that it was impossible to move any of her limbs. This attack came suddenly, without the patient being previously sensible of any pain. Sometimes it lasted from a quarter of an hour to two, three, or even nine hours: it was often accompanied with loquacity, a kind of extatic delirium, of which the patient recollected nothing. She then had excessive pain at her heart, which beat violently. It may be remarked that, some years before, the patient having lost her mother, had similar attacks, but without pain or palpitation. The disease went on increasing. Frequent bleedings, leeches to the precordial region, left leg, neck, and left arm, were used. Every time that the pain came on, baths, stimulating pediluvia, alvine injections, were tried, but without effect. It may be remarked, that leeches always increased her sufferings.

She had been in this way four years when M. Peyron saw her. He discovered, by mediate auscultation, that the beating of the heart was stronger than natural; the pain was referred to the space between the cartilages of the fifth and eighth ribs of the left side; the pulse was frequent, full, and intermittent.

After carefully examining her symptoms, M. Peyron came to the conclusion that it was a case of rheumatism of the heart; and, in that persuasion, acupuncturation was proposed and acceded to by the patient. The girl being placed on her back, leaning to the right side, the first needle, thirteen lines in length, was introduced with a rotatory motion in the space that separates the cartilages of the fifth and sixth ribs, and in a line nearly corresponding to the middle of the cartilage of this last; it was directed towards the heart, going obliquely from below upwards, and from right to left, but without reaching that organ. The patient experienced no pain during the introduction, but, when it was finished, she stretched out her limbs, contracting them with violence during some minutes, without uttering a word, and soon after fell into a delirium similar to that described as resulting from animal magnetism. She *said* she saw distinctly any object, although her eyes were shut, but she always was deceived when she attempted to tell the number of fingers held up before her. She spoke with astonishing volubility, and answered questions in rather an extravagant manner; and, what is remarkable, could not suffer to be touched. This delirium lasted ten minutes: she awoke as from a deep sleep, was fatigued, and did not recollect any thing she had said; she felt severe pain.—A second needle, of fifteen lines, was then introduced in the same intercostal space, corresponding to the sixth rib. Another attack came on : the loquacity was still greater ; she felt no pain, and called out for another needle.—A third acupuncture was made in the same intercostal space, between the two needles already applied, at the spot where the patient complained most of pain, and where the beating of the heart was most sensible. The needle was eighteen lines in length, and was directed from the upper edge of the cartilage of the sixth rib, from above downwards ; it pierced the pericardium, and reached, without doubt, the point of the heart. The sensations it caused were different ; the patient felt a shiver, and the attack soon ceased. This sensation, the length of the needle, and its movements, which followed exactly all those of the heart, sufficiently prove that it was in direct communication with that organ ; and what may add to the conviction is, that the needle was agitated before the intercostal space into which it was introduced had received the impulse of the heart.

From this time the patient felt no more of her accustomed pain ; what she did experience being quite different.

The needles remained nearly forty-eight hours in situ. The puncture of the one last introduced excited much inconvenience : it was the only one that gave any drops of blood ; these flowed rapidly on its extraction, which was very painful. This needle was most oxydised. The patient afterwards felt only a sharp pain at the situation of the punctures, which soon went off, and the rheumatic pain quite disappeared. (*Ibid.*)

---

### SURGERY.

*Suture in Wounds of the Bladder.*—Report of MM. Lisfranc, Manjant, and Amusat, on a Memoir of M. Pinel Grandchamp, entitled "Experiments on Animals, tending to establish the advantages of the Suture to obtain the Reunion of Wounds of the Bladder, and prevent Extravasations of Urine :"—

These experiments may cause the preference to be given to the high operation for lithotomy. The author has, with success, practised sewing up the bladder, after wounding that organ, in dogs, cats, hares, &c.; although in them the wound has always been situated in the most dependent part of the bladder, and of course the urine always in contact with it ; and although the perineum, from its connexions, was cut in two places, which would not happen in man.

In a first series of experiments on twenty-one dogs, twelve were entirely cured, four were healing, and five had perished. In a second series, nine out of ten were cured. In a third, three of seven only were cured ; but in this instance, to render the experiments as nearly alike as possible to what would occur in men about to undergo an operation for the stone, he had introduced into the bladders the fragments of human calculi, pieces of lead, and lumps of gelatine. Now, in two of the dogs, the pieces of lead fixed themselves in the urethra, shut up that canal, and caused accumulation of urine in the bladder, and extravasation of urine in two others. The fragments of the calculi interposed themselves between the points of the suture, and in this way also caused extravasation. The kind of suture used was the Glover's suture, (*la suture du pelletier.*)

M. P. Grandchamp thinks that the employment of this suture would be very applicable in the high operation for the stone.

The reporters have killed several dogs operated on by M. Pinel, in order to discover the state of the parts.

1st. In three dogs operated on within two months, they found perfect cicatrices; one of them a little puckered on the interior; in one also a piece of intestine was adhering to the cicatrix.

2d. On two others, the thread, which had not been removed, formed the nucleus of a calculus; the bladder was thickened and inflamed, and the cicatrix solid.

3d. In one operated on fifteen days, and where the suture had been removed on the third day, the cicatrix was almost complete: the urine passed by the penis.

4th. In one operated on fifteen days, the bladder had adhered to the epiploon; the cicatrix was incomplete, and the urine escaped by the wound.

5th. In another operated on two months, into which a calculus had been introduced, there was a perfect cicatrix in the bladder; but the mucous membrane was red, swollen, and like a sponge at the point where the calculus had rested, and which had increased to three times its original size.

6th. In another operated on fifteen days, the wound in the bladder was cicatrised two-thirds of its extent, and showed a little suppuration: the urine was frothy, and passed by the urethra.

The reporters conclude with M. Pinel, first, that the substance with which the suture is made must be removed, as, without this precaution, it would prove a nucleus for a future calculus; secondly, that, even should the wound of the bladder not be quite cicatrised when the ligature is withdrawn, the bladder contracts adhesions with the parietes of the abdomen, which hinders extravasation; that, in man, the edges of the wound of the abdomen might be kept open, so as to remove the ligature a little later. They conclude, that the experiments of M. Pinel authorise the belief that the process described by that surgeon might be applicable to man.

*Strictures of the Urethra.*—An attentive observation of what occurs in patients affected with this complaint, when they make water, has led Professor Cittadini to an ingenious method of introducing bougies. "When patients with stricture wish to make water, the bladder forcibly expels the urine, which flows to the obstacle: stopped here, it filters, in small quantity at first, through the straitened canal; but it appears that this filtration soon produces a momentary dilatation, since the water comes at times in a full jet, even in patients where no kind of bougie could be introduced." This dilatation from within outwards has often been imitated by the Professor, with the happiest effect, by using injections, and introducing the bougie without permitting the injected fluid to escape.

*Extraction of Calculus from the Female Bladder, by Dilatation of the Urethra.* By Dr. Hamilton.—In November 1823, Mrs. F——, ætatis fifty-five, a stout hale woman from Alloa, presented herself to the New-Town Dispensary, labouring under symptoms of stone. The complaint had existed for six or eight years. Having, by sounding, satisfied myself of the existence of calculus, I informed her that the only relief which could be afforded was by extraction; which would probably be effected by dilatation, without any cutting. She that evening took some opening medicine, and next day, at three P.M., I introduced Mr. Weiss's dilator of the female urethra, and turned the screw up two teeth; so dilating the blades to the extent of one-fourth of an inch. I returned in an hour, and found my patient complaining much of the pain that was produced. A few drops of blood were oozing from the meatus. I turned the screw another point, with great increase of suffering. An opiate was administered. In an hour and a half I again returned; found the oozing of blood more considerable, and the pain more intolerable. It required much persuasion before I was permitted again to turn the screw, and separate the bladder to the fourth division marked on the scale. At two several intervals of an hour and a half, I raised it two other points in the scale, and thus brought the dilation to equal three-fourths of an inch. This, it will be observed, was effected with much severe suffering, in six hours.

At eleven P.M. I withdrew the dilator, and could of course easily introduce the forceps. With a pair of strong polypus forceps I could grasp the stone, but found the hold I maintained was not sufficient. The stone slipt in my endeavours to extract. With a lithotomy forceps, of a small size, I found that the blades were so large and thick that I could obtain no firm grasp; they only rasped along its partially embraced surface.

After satisfying myself that the dilatation was insufficient, I again introduced the instrument, and raised the screw to the seventh point in the scale. A full opiate was administered, and a respite held out, for sleep, during the night.

At seven next morning it was found she had suffered much during a sleepless night. The parts were exquisitely tender, and bleeding a little. The screw was raised another point; and again another, to the ninth, in an hour afterwards. This brought the dilatation to one inch and one-eighth.

I was now convinced that the patience of my patient was completely exhausted. Often had she expressed a wish that the process had never been commenced, and a strong conviction that it was more painful than any cutting. Being, therefore, fully persuaded that she would no longer submit to the further use of the instrument, and solicitous that all her suffering should not be useless, I provided myself with a probe-pointed bistoury, in case the dilatation was not even now sufficient; and at nine A.M. determined, with the assistance of my friend Dr. Molison, to do all in my power for her relief.

A firm hold with the lithotomy forceps could now be procured. The position of the stone could be adjusted in the most favourable manner, and all the force that was regarded safe could be steadily applied. This was done for four or five minutes, but the urethra would not yield. After the dilatation and the force had been carried to what I conceived the extreme limit, I introduced the bistoury, and cut at the angle formed by the junction of the bladder and urethra, in a direction upwards and outwards. The bridle, which had obstructed the progress of the stone, was thus divided, and it was now easily extracted.

The incision must have been to the most trifling extent, as there was no hemorrhage, and the patient was never sensible of any incontinence of urine. Another opiate was administered. The patient lay in bed for the most of the day, and made water at regular intervals. She had no unpleasant symptom whatever after the operation; next day was walking about the house, and in a few days returned home in perfect health. She came to town a few months ago, and called to inform me she was completely cured.

The stone weighed precisely one ounce: its long circumference measures four and a half inches; its short, one inch and a quarter. (*Med.-Chirurgical Transactions of Edinburgh*, vol. ii.)

*Case of Extraction of Calculus from the Female Bladder*. By Dr. Ramsay.—About two weeks were spent, with very partial success, in dilating the urethra by means of sponge tents, which were generally expelled in a few hours after their introduction; but they sometimes remained during the course of a night, though not without much inconvenience and distress. The common polypus forceps, and afterwards a better instrument, (commonly used for extracting balls,) which Dr. Thomson was so obliging as to send me, were tried with better effect. But in Weiss's dilator I found an instrument well suited to its purpose, and succeeded in expanding the urethra more and more every day, from twenty to thirty minutes at a time, until, in the course of ten days, I could easily introduce the forefinger within the urethra, and examine with sufficient accuracy the figure, size, and surface of the calculus.

Prior to extraction, I introduced the dilator, in the presence of Mr. Crichton and Dr. Nimmo, and turned the screw very gradually until, in the course of about thirty minutes, I had opened the blades sufficiently to admit the flat side of the forceps within them. The dilator was then withdrawn. I fortunately seized the stone in the best manner for its extraction, which was accomplished with some difficulty, and a good deal of pain to the patient. The chief resistance was found at the orifice of the urethra, and was at first so great as to render it doubtful how far the bistoury might not have been advisable; but, as this might have defeated the great object in view, I persisted a little longer, and effected the extraction without its aid.

From the size of the stone, (which we found to be five inches and a quarter in its long circumference, and three and a half inches in the short, and weighing seven and a half drachms,) I apprehend it would have been impossible to have extracted it in this way, without rupturing the urethra, had not the dilatation been effected in a very *gradual* but *decided* manner for some weeks previous to the operation.

A good deal of general irritation prevailed in the system for a few days after the removal of the stone, with pain and swelling of the parts; but these symptoms soon gave way,—not, however, without considerable mucous and puriform discharge from the vagina, with some membranous sloughs, which seemed to give considerable uneasiness by obstructing the free passage of urine.

The bladder and urethra very quickly resumed their natural functions. The power of retention, which from the first was never lost, has ever since remained perfect; and all the distressing symptoms depending upon stone in the bladder, entirely left her after the operation. (*Ibid.*)

*Chlorate of Lime in Burns.*—M. Lisfranc has been for some time in the habit of applying this active substance in burns, and with great success. Several cases are published in the account of the Clinique of La Pitié, where the application of the substance was made immediately after the accident had occurred; occasionally, emollient cataplasms were premised. M. L. at first dreaded the production of too violent an inflammation from its direct application; but accident having shown him the contrary in a very desperate case that occurred, he now has recourse to it immediately. This chlorate of lime ought to be in strength equal to 3° of M. Gay Lussac's chlorometre. When used, the dressing of the wound is made in this way: the part burnt is to be covered with lint, with holes in it, and spread with cerate; above this is put a great quantity of charpie, wet with the solution, and this is to be kept constantly moistened with it. M. Lisfranc has great confidence in this application.

